# Stream Segregation in the Perception of Sinusoidally Amplitude-Modulated Tones

**DOI:** 10.1371/journal.pone.0043615

**Published:** 2012-09-12

**Authors:** Lena-Vanessa Dolležal, Rainer Beutelmann, Georg M. Klump

**Affiliations:** Animal Physiology and Behavior Group, Department of Biology and Environmental Sciences, Carl von Ossietzky University Oldenburg, Oldenburg, Germany; Max Planck Institute for Human Cognitive and Brain Sciences, Germany

## Abstract

Amplitude modulation can serve as a cue for segregating streams of sounds from different sources. Here we evaluate stream segregation in humans using ABA- sequences of sinusoidally amplitude modulated (SAM) tones. A and B represent SAM tones with the same carrier frequency (1000, 4000 Hz) and modulation depth (30, 100%). The modulation frequency of the A signals (*f_modA_*) was 30, 100 or 300 Hz, respectively. The modulation frequency of the B signals was up to four octaves higher (Δ*f_mod_*). Three different ABA- tone patterns varying in tone duration and stimulus onset asynchrony were presented to evaluate the effect of forward suppression. Subjects indicated their 1- or 2-stream percept on a touch screen at the end of each ABA- sequence (presentation time 5 or 15 s). Tone pattern, *f_modA_*, Δ*f_mod_*, carrier frequency, modulation depth and presentation time significantly affected the percentage of a 2-stream percept. The human psychophysical results are compared to responses of avian forebrain neurons evoked by different ABA- SAM tone conditions [1] that were broadly overlapping those of the present study. The neurons also showed significant effects of tone pattern and Δ*f_mod_* that were comparable to effects observed in the present psychophysical study. Depending on the carrier frequency, modulation frequency, modulation depth and the width of the auditory filters, SAM tones may provide mainly temporal cues (sidebands fall within the range of the filter), spectral cues (sidebands fall outside the range of the filter) or possibly both. A computational model based on excitation pattern differences was used to predict the 50% threshold of 2-stream responses. In conditions for which the model predicts a considerably larger 50% threshold of 2-stream responses (i.e., larger Δ*f_mod_* at threshold) than was observed, it is unlikely that spectral cues can provide an explanation of stream segregation by SAM.

## Introduction

The auditory system uses spectral or temporal cues to integrate or segregate signals to form auditory streams in the analysis of acoustic scenes (auditory stream segregation, [2]). Most natural signals provide a mixture of spectral and temporal cues. Sinusoidally amplitude modulated (SAM) tones being used in the present study as a model for the processing of natural signals in auditory stream segregation have the advantage that the carrier frequency, the modulation frequency and the modulation depth can be adjusted in such a way that, depending on the parameter values and the auditory filter bandwidth [3], SAM tones provide either temporal cues or spectral cues or both. This variation of parameter values allows evaluating the relevance of the different cues for stream segregation.

Sequentially presented signals are segregated into different auditory streams if acoustic differences between signals are perceptually salient and are integrated into one auditory stream if these differences are small or even diminished. Moore and Gockel [4] concluded that “… the extent to which sequential stream segregation occurs is directly related to the degree of perceptual difference between successive sounds”. How the different cues affect auditory stream segregation can be analyzed by applying the commonly used ABA- paradigm [5]. The ABA- paradigm consists of sequentially presented A and B signals. ABA- sequences can either be integrated and perceived as one single galloping rhythm (ABA-ABA-ABA-… i.e. one stream) or separated into simultaneous sequences one with a fast and one with a slow isochronous rhythm (A-A-A-A-A-A-… and -B—B—B—B–…, i.e., two streams), where the dash indicates a silent interval of the same duration as the tone duration.

The present study using the ABA- paradigm has two major goals: Firstly, psychophysical data will be compared to neurophysiological responses observed previously [1] to evaluate whether the neuronal response patterns are indicative of the mechanisms underlying stream segregation by SAM. Secondly, the relevance of spectral and temporal cues for stream segregation by SAM will be evaluated by applying a computational model of auditory processing to explain stream segregation perceived by human subjects. In the following we will develop predictions addressing both topics.

### Comparison of perception with neuronal responses

The auditory systems of European starlings and humans have similar temporal and spectral resolution (e.g., [6]). Thus, it can be expected that human perception in an auditory stream segregation paradigm using similar SAM tones as in the physiological study [1] shares many features with the pattern of activity of starling auditory forebrain neurons. Therefore, the present study chose to present a set of SAM tone stimuli in an ABA- paradigm in which stimuli were varied across a comparable range of parameters. In the European starling Itatani and Klump [1] observed a neural correlate of auditory stream segregation of SAM tones with the same carrier frequency but different modulation frequencies. In the present psychophysical study with human subjects we used carrier frequencies of 1000 and 4000 Hz (starling range 400 to 6000 Hz). The modulation frequency of the A SAM signal ranged from 30 to 300 Hz (starling≤160 Hz) and the modulation frequency of the B signal was up to 4 octaves above the modulation frequency of the A signal (both human and starling). Previous psychophysical studies in humans [5], [7]–[8] and neurophysiological studies in starlings [9]–[11] have evaluated the effect of tone duration (TD) of pure tones on auditory stream segregation. In their first experiment, Bregmann et al. [7] presented ABA- sequences with a constant stimulus onset asynchrony (SOA_across_; onset-to-onset time between two successive tones) of 100 ms and provided evidence that an increase in TD from 40 to 140 ms increased the percentage of a 2-stream percept. Beauvois [8] presented ABAB sequences and used slightly different stimulus parameters (SOA_across_ = 130 ms, TD≤120 ms) and obtained results comparable to those reported by Bregman et al. [7] in their first experiment. Van Noorden [5] presented random sequences of tones having varying TD (80 to 400 ms) and correspondingly varying SOA_across_ (SOA_across_ = TD) and observed that an increase in TD reduced the percentage of a 2-stream percept. Bregmann et al. ([7]; 2^nd^ experiment) presented ABA- sequences and observed, like van Noorden [5], a reduced percentage of a 2-stream percept for pure tones with increasing TD of 75–150 ms and correspondingly varying SOA_across_ (SOA_across_ = TD). In starling physiology the influence of the TD was studied by presenting abutting A and B signals with correspondingly varying SOA_across_ (SOA_across_ = TD; [9], [10]) or by presenting ABA-signals with a constant SOA_across_ but varying TD [9], [11]. In general, in the starling the effect of TD in ABA-sequences with varying SOA_across_ (SOA_across_ = TD) was either not significant [9] or indicated a reduction [10] in the proportion of neuronal 2-stream responses with increasing TD (25–100 ms). Neurophysiological studies presenting ABA- stimuli with increasing TD but constant SOA_across_ showed either no effect of TD [9] or indicated an increase in the neuronal response differences (comparable to an increase in 2-stream responses) for ABA-sequences of longer TD (100 ms) than to sequences presented with shorter TD (25 ms; [11]). The psychophysical auditory streaming studies regarding the effect of the timing parameters (e.g.; varying TD and SOA_across_) so far have primarily focused on tones rather than complex stimuli. Therefore, we presented SAM tones in ABA-sequences having two different TD (125, 375 ms) to the human subjects. For SAM tone stimuli with a TD of 125 ms that were presented both to humans and starlings [1], we evaluated the effect of varying SOA_across_ (125, 375 ms in humans; 125, 250 or 500 ms in starlings). This analysis of the effect of constant TD and varying SOA_across_ can provide evidence for suppression between sequential signals. In addition the effect of varying TD and constant SOA_across_ (125 ms/375 ms and 375 ms/375 ms) and the effect of varying TD and varying SOA_across_ (TD = SOA_across_; 125 ms/125 ms and 375 ms/375 ms) was evaluated and compared to previously published results [1], [5], [7]–[11].

Since there was a broad overlap of the SAM tone parameter conditions presented to humans and starlings, we can make use of the physiological results to predict the outcome of the psychophysical experiments. Since in the physiological study by Itatani and Klump [1] the spike rate dropped significantly at modulation frequency differences larger than 1 octave, we predict a significantly larger percentage of a 2-stream percept beginning at this modulation frequency difference between the A and the B SAM tone. Furthermore, based on the physiological data indicating forward suppression effects [1] comparable to those observed in pure tones [9], [11] we predict a lower percentage of a 2-stream percept for the SAM tone pattern of 125 ms/375 ms (TD/SOA_across_) than for the SAM tone pattern of 125 ms/125 ms (TD/SOA_across_). In addition to the comparison with the neurophysiological study [1], the present study will discuss the results with reference to other psychophysical [3], [5], [7]–[8], [12]–[14] and neurophysiological studies [9]–[11], [15]–[17].

### The relevance of spectral and temporal cues

Streaming based on spectral cues, e.g., pure tone frequency, has been explained by the peripheral channeling hypothesis [18]–[19]. This hypothesis predicts that signals are more likely to be integrated if they excite auditory filters that cover a similar spectral range than if they excite auditory filters that cover a spectral range that is well separated. Results from physiological studies using pure tones are compatible with the peripheral channeling hypothesis (e.g., [9], [11], [16]–[17], [20]). Even for two-tone complexes Cusack and Roberts [21] observed the smallest perceptual segregation for signals with similar bandwidth supporting the idea of the importance of filter bandwidth for auditory stream segregation. A perceptual segregation of streams, however, can also occur if the stimuli do not differ in their spectral pattern of excitation. It has been demonstrated in psychophysical [12], [22]–[24] and physiological studies [1], [24]–[25] that temporal cues alone can be sufficient for stream segregation. By exploring an even larger parameter space than was presented in the physiological study by Itatani and Klump [1], the present study evaluates in which SAM tone conditions spectral or temporal cues are more likely to contribute to the perceptual segregation of streams. Mainly temporal cues are provided by SAM tones with a high carrier frequency and a low modulation frequency, whereas spectral cues are elicited at a low carrier frequency and a high modulation frequency. The spectral change of excitation may be detected if the sideband frequencies fall outside the range of the auditory filter centered at the carrier frequency (e.g., [3], [13]). To evaluate whether spectral cues provide sufficient information for auditory stream segregation of SAM tones differing in modulation frequency or whether temporal cues must be involved, a computational model based on excitation pattern differences in the auditory periphery was developed to predict the 50% threshold of 2-stream responses using the approach pursued by Dau et al. [26]–[27]. In conditions in which the computational model calculated a similar or a smaller 50% threshold of 2-stream responses (i.e., smaller or similar Δ*f_mod_* at threshold) than the perceptually measured 50% threshold of 2-stream responses spectral cues are likely to be sufficient for stream segregation. In conditions, however, in which the calculated 50% threshold of 2-stream responses is considerably larger (i.e., larger Δ*f_mod_* at threshold) than the perceptually measured 50% threshold of 2-stream responses spectral cues are unlikely to explain perceptual stream segregation. In such SAM tone conditions the perceptual 50% threshold of 2-stream responses is more parsimoniously explained by temporal cues.

## Materials and Methods

### Ethics Statement

The experiments were undertaken with the understanding and written consent of each subject, following the Code of Ethics of the World Medical Association (Declaration of Helsinki). The experiments were approved by the local ethics committee of the University of Oldenburg.

### Subjects

Eight human subjects (one male and seven females, including the first author) with the mean age of 27.6±2.3 (SEM) were tested. Seven subjects participated in the main experiment and three subjects (including two from the main experiment) participated in the control experiment. The main experiment evaluated the perceptual segregation of SAM tones using the ABA- paradigm varying the stimulus parameters as described below. The control experiment served to evaluate whether distortion products may have provided additional spectral cues for the auditory system to segregate streams. All participating subjects had normal hearing defined as pure-tone thresholds of less than 20 dB hearing level. Four of the subjects had previous experience with psychophysical experiments. Prior to the experiment each subject attended training sessions to become familiar with the test paradigm. In these sessions all ABA- sequences with the different combinations of parameters were presented once.

### Acoustic stimuli and conditions

In this study the ABA- sequences consisted of sinusoidally amplitude modulated (SAM) A and B signals. The SAM tones (10 ms raised cosine rise/fall) were produced by a Hammerfall DSP (Multiface II, RME) and presented diotically with calibrated headphones (Sennheiser HDA 200) in a sound-attenuating chamber (IAC, Industrial Acoustics Company, Mini 250). The SAM tones were computer generated (Matlab, Version 7.1) at a sampling frequency of 44100 Hz by the following function:
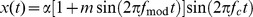

*x(t)*: A or B signal

*α*: scaling factor used to set a constant overall level

*m*: modulation index

*f_mod_*: modulation frequency

*f_c_*: carrier frequency

In this study the modulation depth of the SAM tones was either 30% (*m* = 0.3) or 100% (*m* = 1.0). The reference modulation frequency of the A signal (*f_modA_*) was fixed at 30, 100 or 300 Hz (i.e., constant within a session), whereas the modulation frequency of the B signal was varied in 0.5 octave steps and was up to four octaves higher (Δ*f_mod_*) than *f_modA_*. Consequently, the SAM frequency of the B signal was always above that of the A signal. The A and B signals had the same carrier frequency (*f_c_*) of 1000 or 4000 Hz, respectively. The ABA- triplets were repeated for a presentation time of 5 or 15 s to explore whether a longer presentation of the ABA- sequence of SAM tones results in increased stream segregation indicating a build-up effect. The amplitude of each SAM tone was adjusted to an overall presentation level of 70 dB SPL by varying *α* depending on the modulation depth. SAM tones have a three-line spectrum. The central spectral peak represents the carrier frequency and the frequencies of the two sidebands, one on each side, are separated from the carrier frequency by the modulation frequency (*f_mod_*). At a modulation depth of 100% the level of the carrier alone was 68.2 dB SPL, whereas the level of each of the corresponding sidebands was 62.2 dB SPL. At the lower modulation depth of 30% the level of the carrier alone was 69.8 dB SPL, whereas the level of each of the corresponding sidebands was 53.3 dB SPL.

ABA- sequences with three different tone patterns were presented in order to examine the effect of the tone duration (TD) and stimulus onset asynchrony (SOA_across_; onset-to-onset time between two successive tones) on the perceptual stream segregation as shown in studies presenting pure tones (e.g., [5], [7]–[11]) and in the physiological study presenting SAM tones [1]. The three tone patterns presented here consist of specific combinations of TD and SOA_across_. The standard SAM tone pattern refers to an ABA- sequence with widely spaced short signals (shortly denoted as 125 ms/375 ms referring to a TD of 125 ms and a SOA_across_ of 375 ms). The other two presented tone patterns are transformations of the standard with either the same TD but a shorter SOA_across_ (125 ms/125 ms) or with the same SOA_across_ than the standard tone pattern but a longer TD (375 ms/375 ms).

In addition to the main experiment, a control experiment was conducted to evaluate whether distortion products resulting from the interaction of the spectral components of the SAM tones (e.g., [28]) may have affected the perceptual segregation. To mask any distortion products, ABA- sequences were presented in a continuous background of pink noise in the control experiment. Pink noise was chosen to mask distortion products especially in the low frequency range (e.g., [28]–[30]). The pink noise masker was computer-generated (Matlab, Version 7.1), had a slope of-3 dB/oct above 100 Hz with an upper cut-off frequency of 22050 Hz and a constant power spectral density below 100 Hz. The overall level of the noise was 70 dB SPL. The masking noise level per critical band was at least 52 dB SPL. The control experiments were conducted for all SAM tone conditions of a modulation depth of 100%. The level of the masking noise was sufficiently large to mask any distortion products due to the interaction between the spectral components of the carrier frequency and the two sidebands (spectral components of the modulation frequency; see Hauser and Probst [31]). For evaluating the effect of possible distortion products the percentage of a 2-stream percept was compared in conditions with and without the pink-noise background.

### Procedure

To measure the perceptual segregation of the successive A and B SAM signals, subjects were instructed to indicate their percept (one or two streams) at the end of each presented ABA- sequence on a touch screen (Elo, 1542L, 15″, Rear-Mount Touchmonitor). After the subjects indicated their percept, a new trial was initiated. To estimate the percentage of a 2-stream percept, each parameter condition was presented six times per subject and the average percentage of a 2-stream percept was calculated. During each test session the carrier frequency, the modulation depth, the Δ*f_mod_* and the presentation time of the ABA- SAM sequence were randomized. Within one test session the tone pattern and the *f_modA_* were constant.

Subjects participating in the control experiment always participated first in the main experiment (ABA- sequences without the noise; one subject listened to the ABA- sequences with 100% modulation depth, only and was thus not included in the analysis of the main experiment). For the control experiment the subjects were instructed to concentrate on the ABA- sequence, as in the main experiment (without noise) and to treat the noise as a background. Apart from the added background noise the experimental design and procedure was the same as in the main experiment.

### An excitation pattern model for evaluating the relevance of spectral or temporal cues for stream segregation of SAM tones

According to the peripheral channeling hypothesis [18], the percentage of a 2-stream percept in an ABA- sequence should increase with increasing difference between the spectral excitation patterns elicited by A and B tones. We used an established model of the auditory periphery [26]–[27] to generate spectral excitation patterns of ABA- triplets individually for each presented SAM tone condition. It is assumed that differences in excitation patterns predicted by the peripheral model relate to differences in the percentage of a 2-stream percept observed for ABA- triplets. Based on this assumption the model generates a psychometric function relating differences in the modulation frequency of the second signal in the triplet to differences in the percentage of a 2-stream percept. In order to compare measured psychophysical and modeled data, the measured 50% thresholds of 2-stream responses
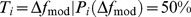
were calculated for each parameter condition 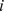, with octave separation Δ*f_mod_* and percentage of 2-stream responses 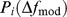 using the psychophysical data of ABA- sequences of a presentation time of 5 s. The thresholds were determined by interpolating linearly between the nearest measured percentages above and below 50%, or by extrapolating linearly from the two percentages directly above 50% in cases in which the percentage of 2-stream responses at the lowest measured octave separation (0.5 octaves) was already above 50%. The predicted thresholds 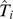 were derived from excitation patterns computed by the model [26]–[27] as described in detail below. For each parameter condition the modeled excitation provided by the B tone, 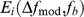 with auditory filter center frequencies 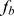, was subtracted in each filter from the corresponding reference excitation provided by the second SAM tone in the triplet with a Δ*f_mod_ of 0* octaves (i.e., the second SAM tone in an AAA- sequence) and integrated across frequency in order to yield a single overall difference value for each condition 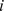 and octave separation Δ*f_mod_*:
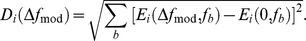
The predicted thresholds
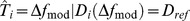
were calculated with a single reference value 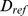 for all conditions. The reference value was obtained by fitting the model data to the measured data by minimising the mean squared error between model and measurement at the 50% thresholds in the three conditions with a carrier frequency of 1000 Hz, an *f_modA_* of 300 Hz, and a modulation depth of 100%. These conditions were chosen as a reference because they provide the largest spectral differences in the excitation patterns elicited by A and B signals since in these conditions the spectral components of the carrier and the sidebands are clearly resolved by the auditory periphery and thus do not provide substantial temporal cues (e.g., [32]).

The model used for calculating the excitation patterns consists of the following components: The stimulus waveform was first filtered by a first order Butterworth band-pass filter with a lower cut-off frequency of 1000 Hz and an upper cut-off frequency of 4000 Hz approximating the transfer function of the middle ear [33]. The middle ear band-pass filter was followed by a linear gammatone filter bank [34] modeling the spectral analysis of the basilar membrane. The filters of the gammatone filter bank had a bandwidth of one ERB [35]. The filters' center frequencies ranged from 100 to 10000 Hz with a constant spacing of two filters per ERB. They were chosen such that one of the filters had a center frequency at the carrier frequency of the SAM signals. The output signals of each of the filters were processed according to Dau et al. [26]–[27], i.e., were first half-wave rectified and filtered by a first-order low-pass filter with a cut-off frequency of 1000 Hz. This stage coarsely represents inner hair cell mechanisms including the loss of phase locking towards high frequencies. It is followed by an adaptation stage which simulates the adaptive properties of the synapse between inner hair cells and the auditory nerve. The adaptation stage consists of a sequence of five feedback loops with different time constants that transforms stationary input signals to a roughly logarithmic scale while fast fluctuations are passed through almost linearly (for details see Dau et al. [26]–[27]). This leads to simulated temporal patterns of excitation similar to those measured in the auditory nerve including a strong peak at the onset of an input signal, decay to a sustained part and an undershoot below the resting state after the offset of an input signal. While the pattern qualitatively resembles the instantaneous firing rate of an auditory nerve fiber, its magnitude is expressed in relative “model units” (MU), which approximate a dB scale for the sustained part of a processed stimulus. The simulation calculated the response for each SAM tone in the ABA- triplet. The mean excitation during presentation of the B tone (without pauses) was compared to the mean excitation during presentation of the second A tone in an AAA- sequence as described above.

### Statistical analysis

The percentage of a 2-stream percept in relation to Δ*f_mod_* (in octaves), *f_modA_*, modulation depth, carrier frequency, presentation time and tone pattern was analyzed using Generalized Linear Mixed Model (GLMM ANOVA, SPSS Statistics Version 17.0). Both the main effects and the two-way interactions were considered. A similar analysis of the main effects was conducted for the control experiment. For comparing the results of the control experiment to the main experiment, we included the presentation of the noise as an additional factor in the GLMM ANOVA. Results from subsequent pair-wise comparisons were Bonferroni corrected.

## Results

### Perceptual segregation of SAM tones

**Figure 1:**
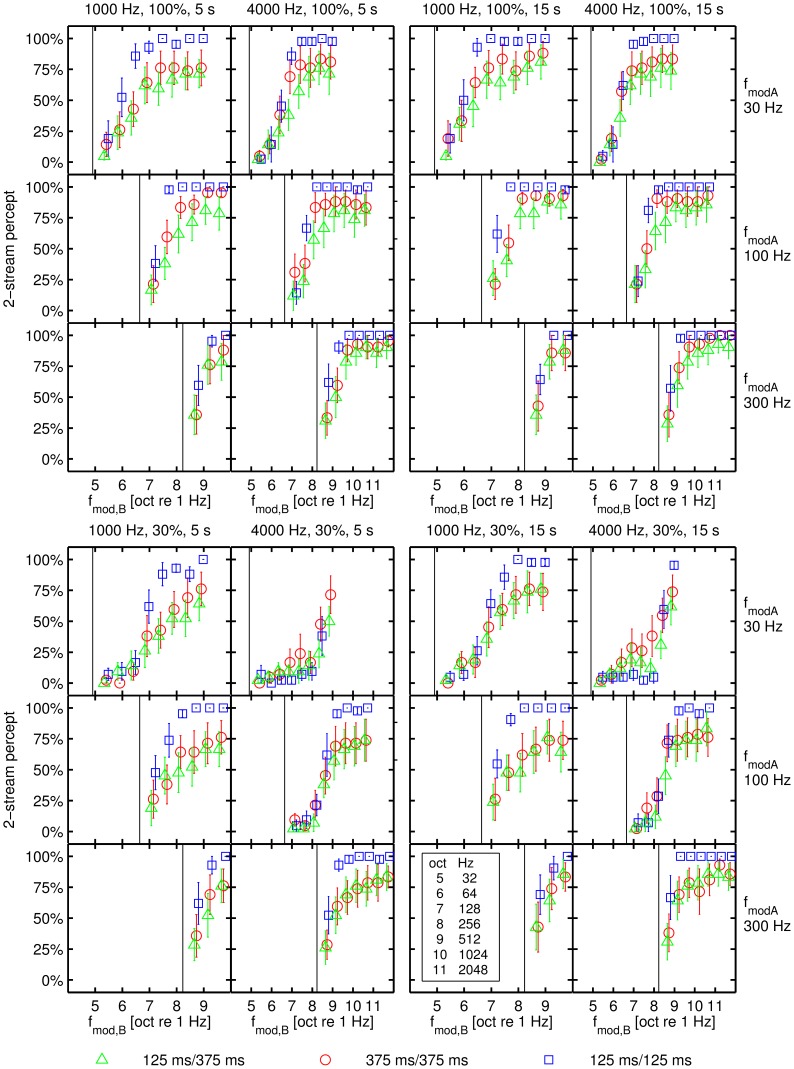
The percentage of a 2-stream percept in relation to the modulation frequency of the B SAM tone. The percentage of a 2-stream percept depicted in relation to the modulation frequency of the B SAM tone in octaves (with reference to 1 Hz). Each of the four blocks represent data for a specific modulation depth (%) and presentation time (s). The two columns within each block represent data for different carrier frequencies (Hz). A vertical black line indicates the position of the reference modulation frequency *f_modA_*, which is also indicated on the right hand side of each row of panels. The error bars denote the standard error and the coloured symbols indicate data obtained for the three different tone patterns (shortly denoted as TD/SOA_across_).

The percentage of a 2-stream percept evoked by SAM tones having the same carrier frequency but different modulation frequencies was analyzed for seven human subjects. With increasing Δ*f_mod_* between the A and the B SAM tones the percentage of a 2-stream percept increased. Figure 1 illustrates the mean percentage of a 2-stream percept (SEM was computed by first averaging across repetitions for each individual subject and then calculating the mean and SEM across subjects) in relation to the modulation frequency of the B SAM tones in octaves (with reference to 1 Hz). An analysis of the main effects in a GLMM model provided significant effects of *f_modA_*, Δ*f_mod_* (in octaves), carrier frequency, tone pattern, modulation depth (*m*) and presentation time (Tab. 1). Furthermore, many two-way interactions were significant.

**Table 1:** Results of the statistical analysis of the percentage of a 2-stream percept.

Effect	F	*p*
f_modA_	377.85	<0.001
Δf_mod_	257.02	<0.001
f_c_	69.53	<0.001
tone pattern	232.38	<0.001
m_depth_	218.15	<0.001
presentation time	25.23	<0.001
f_modA_×Δf_mod_	9.22	<0.001
f_modA_×f_c_	11.27	<0.001
f_modA_×tone pattern	5.44	<0.001
f_modA_×m_depth_	69.56	<0.001
f_modA_×presentation time	0.20	= 0.816
Δf_mod_×f_c_	2.50	= 0.015
Δf_mod_×tone pattern	0.91	= 0.548
Δf_mod_×m_depth_	15.84	<0.001
Δ_fmod_×presentation time	0.30	= 0.956
f_c_×tone pattern	17.69	<0.001
f_c_×m_depth_	53.14	<0.001
f_c_×presentation time	0.05	= 0.822
tone pattern×m_depth_	2.24	= 0.107
tone pattern×presentation time	1.36	= 0.256
m_depth_×presentation time	0.51	= 0.476

Results of a GLMM analysis of the percentage of a 2-stream percept in relation to the stimulus parameters *f_mod, A_*, Δ*f_mod_*, carrier frequency, tone pattern, modulation depth and presentation time. F- and p-values for main effects and two-way interactions are displayed. Bold numbers highlight the significant effects.

The percentage of a 2-stream percept increased with increasing *f_modA_* for the same change of Δ*f_mod_* (in octaves; Fig. 1). ABA- sequences with an *f_modA_* of 30 Hz evoked the least percentage of a 2-stream percept (mean = 47.7%) which was significantly less than the percentages of a 2-stream percept evoked by ABA- sequences with an *f_modA_* of 100 Hz (mean = 69.9%; *p*<0.001) and 300 Hz (mean = 81.8%; *p*<0.001). In addition the percentage of a 2-stream percept of an *f_modA_* of 100 Hz differed from the percentage of a 2-stream percept of an *f_modA_* of 300 Hz (p<0.001). In general, up to a Δ*f_mod_* of 2.5 octaves each increase of Δ*f_mod_* by 0.5 octaves resulted in a significant increase of the percentage of a 2-stream percept (*p*≤0.049). At a larger Δ*f_mod_* the percept stabilized and reached a constant value (ceiling effect) with the exception of the carrier frequency of 4000 Hz and the modulation depth of 30%. For this parameter condition the percentage of a 2-stream percept had not reached a plateau within the tested range of Δ*f_mod_* in octaves (Fig. 1). SAM tones with a carrier frequency of 1000 Hz were more likely to evoke a 2-stream percept (mean = 71.3%) than SAM tones with a carrier frequency of 4000 Hz (mean = 60.3%; *p*<0.001). Especially at an *f_modA_* of 30 Hz, a difference in the percentage of a 2-stream percept for SAM tones differing in the carrier frequency (mean 1000 Hz = 55.4%; mean 4000 Hz = 40.1%) was observable (Fig. 1).

A strong effect of the tone pattern on the percentage of a 2-stream percept was observed (Fig. 1; Tab. 1). In agreement with the results of the study by Itatani and Klump [1] the standard tone pattern with widely spaced short signals (125 ms/375 ms) evoked the least segregated percept (mean = 55.4%) compared to the tone pattern with abutting short signals (125 ms/125 ms; mean = 79.4%; *p* = 0.004). The mean percentage of a 2-stream percept elicited by the tone pattern with abutting long signals (375 ms/375 ms; mean = 62.6%), however, was smaller than the percentage of a 2-stream percept of ABA- sequences with abutting short signals (125 ms/125 ms; *p* = 0.048). ABA- sequences of SAM tones with a modulation depth of 30% (mean = 58.1%) are perceptually less segregated than SAM tones with a modulation depth of 100% (mean = 73.5%; *p*<0.001). The percentage of a 2-stream percept is most strongly affected by the modulation depth at an *f_modA_* of 30 Hz (*m* = 30%, mean = 34.0%; *m* = 100%, mean = 61.5%). The effect of the modulation depths on the percentage of a 2-stream percept was reduced with increasing *f_modA_* (Fig. 1). At an *f_modA_* of 300 Hz the percentage of a 2-stream percept for the two modulation depths is almost identical (*m* = 30%, mean = 80.5%; *m* = 100%, mean = 83.1%). An increase of the presentation time from 5 s to 15 s increased the subjects' percentage of having a 2-stream percept (presentation time = 5 s, mean = 63.4%; presentation time = 15 s, mean = 68.2%; *p*<0.001; Fig. 1), as expected from the build-up effect observed in other studies (e.g., [10], [36]–[37]).

### Control experiment: Possible Effects of Distortion Products

**Figure 2:**
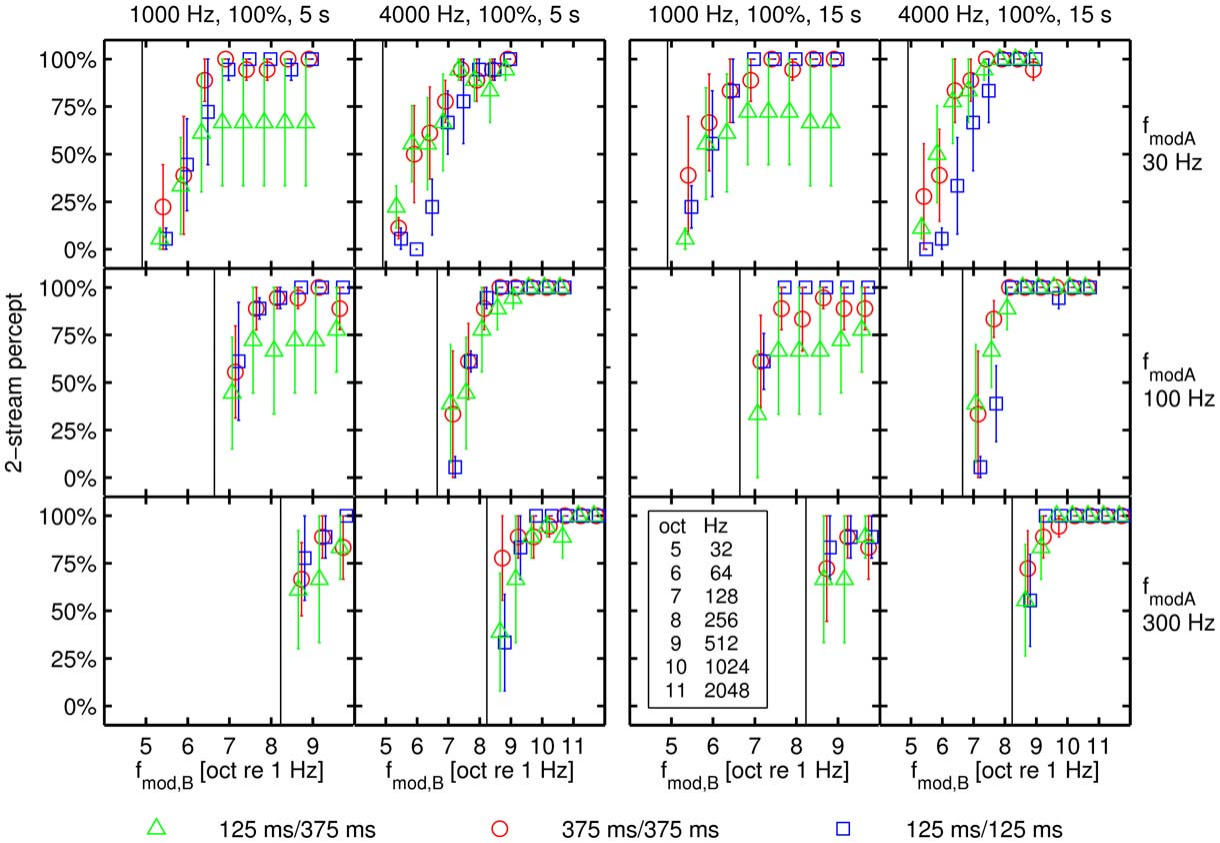
The percentage of a 2-stream percept in relation to the modulation frequency of the B SAM tone while a continuous pink noise masker is presented. The percentage of a 2-stream percept depicted in relation to the modulation frequency of the B SAM tone in octaves (with reference to 1 Hz). The two blocks represent data for a modulation depth of 100% with two different presentation times (5 and 15 s, respectively). The two columns within each block represent data for the different carrier frequencies (Hz). A vertical black line indicates the position of the reference modulation frequency *f_modA_*, which is also indicated on the right hand side of each row of panels. The error bars denote the standard error and the coloured symbols indicate data obtained for the three different tone patterns (shortly denoted as TD/SOA_across_).

In the control experiment a continuous pink noise being presented in the background of the ABA- SAM sequences served to mask possible distortion products that may arise from the interaction of spectral components of the SAM tones. Similar to the main experiment, the mean percentage of a 2-stream percept (SEM was computed by first averaging across repetitions for each individual subject and then calculating the mean and SEM across subjects) started from the lower baseline and increased in all parameter conditions with an increasing modulation frequency difference between the A to the B SAM tone (Fig. 2). Over all tested conditions, the percentage of a 2-stream percept was larger in the control experiment (mean = 79.0%; Fig. 2) than the percentage of a 2-stream percept evoked in the main experiment without the masking noise (mean = 75.4%; *p* = 0.003, Fig. 1). The significant effects however were comparable for the main and the control experiment with two exceptions. In the control experiment there was no significant main effect of carrier frequency (mean 1000 Hz = 78.5%; mean 4000 Hz = 81.0%) and presentation time (presentation time = 5 s, mean = 78.4%; presentation time = 15 s, mean = 81.1%).

### Predicting the perceptual segregation of SAM tones by an excitation pattern model

**Figure 3:**
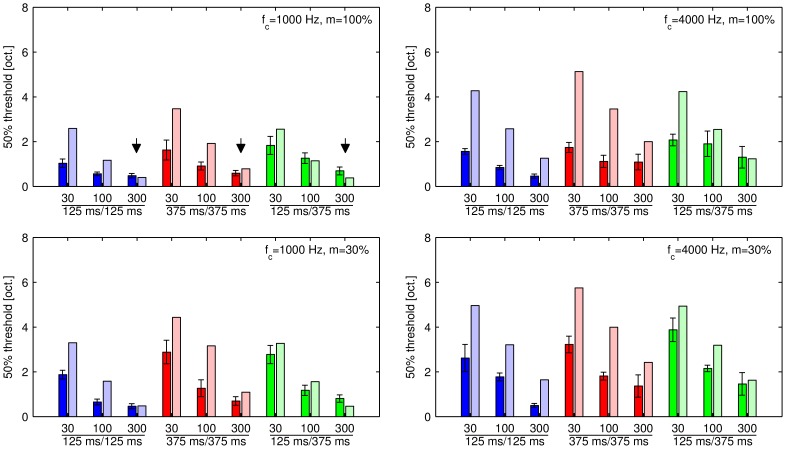
The subjects' mean 50% thresholds of 2-stream responses and corresponding modelled excitation patterns. The subjects' mean 50% thresholds of 2-stream responses calculated from observed data (dark colours) and modelled excitation patterns (pale colours). The panels show data for different carrier frequencies (*f_c_* in columns) and modulation depths (*m* in rows). The data is grouped by tone pattern (shortly denoted as TD/SOA_across_; colours), and labelled with the reference modulation frequency in Hz within each group. The error bars denote the standard error. The arrows mark the three conditions used to fit the model data to the observed data.

**Figure 4:**
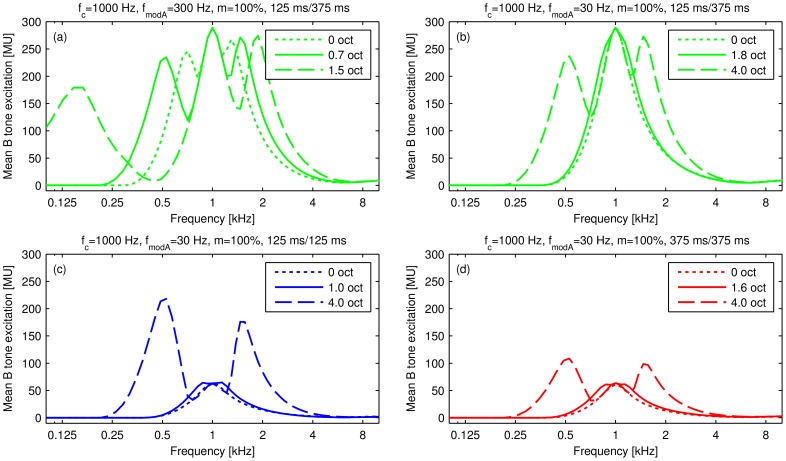
Modelled spectral excitation patterns for selected conditions. Modelled spectral excitation patterns in arbitrary “model units” (*MU*) for selected conditions (parameters given in the panel titles). Each panel shows, for the respective condition, one excitation pattern (dotted line) at the reference modulation frequency (*f_modA_*), one excitation pattern (solid line) at the 50% threshold of 2-stream responses calculated from the measured data (values given in the legends) and one excitation pattern at the maximal octave separation measured (dashed line). The excitation patterns reflect the mean model activation during presentation of the B tone excluding the silent gaps occurring in some of the conditions. The different colours indicate the tone pattern (shortly denoted as TD/SOA_across_) as in the other figures.

A computational model [26]–[27] was used to calculate the 50% thresholds of 2-stream responses based on the differences between the spectral excitation pattern elicited by the second SAM tone in the triplet with a Δ*f_mod_* of 0 octaves (reference) and the second SAM tone in the triplet with a larger Δ*f_mod_* of up to 4 octaves. These thresholds were compared to the measured psychophysical 50% thresholds of 2-stream responses (Fig. 3). The model reference value *D_ref_* was fitted to a subset of the measured response data minimizing the sum-of-least-squares difference for the three parameter conditions indicated by the arrows (Fig. 3, *f_c_* = 1000 Hz, *f_modA_* = 300 Hz) for which the sidebands are resolved by the auditory filters (which makes the use of temporal modulation cues less likely) and that provide for the largest spectral excitation pattern differences at the 50% threshold of 2-stream responses (for a comparison of these patterns see Fig. 4a). For all other conditions, the reference value *D_ref_* obtained with the excitation patterns differences at a carrier frequency of 1000 Hz and an *f_modA_* of 300 Hz was used to predict the thresholds. In general, the model predictions increase with decreasing *f_modA_* in all conditions which is consistent with the measured psychophysical data (Fig. 3). The predicted 50% threshold of 2-stream responses in many conditions, however, was well above the measured psychophysical threshold. Especially in conditions in which the sidebands created by the modulation frequency lay within the limits of the auditory filter centered at the carrier frequency (e.g., *f_c_* = 4000 Hz, *f_modA_*≤100 Hz) the model predictions were considerably higher than the measured threshold data (Fig. 3). Assuming that similarly large differences between the excitation pattern elicited by the second SAM tone in the triplet with a Δ*f_mod_* of 0 octaves (reference) and the second SAM tone with a larger Δ*f_mod_* of up to 4 octaves define the 50% threshold of 2-stream responses based on spectral differences in all conditions, predicted thresholds that are considerably higher than measured thresholds suggest that spectral patterns of excitation are unlikely to provide sufficient cues for stream segregation. The computational model based on the excitation pattern differences, however, was not able to predict any of the low 50% thresholds of 2-stream responses for the control experiment.

Examples of excitation patterns for the main experiment predicted by the computational model for the A SAM tone modulation frequency (dotted line), the B SAM tone modulation frequency at the 50% threshold of 2-stream responses (solid line), and for the highest modulation frequency of the B SAM tone that was tested (dashed line; the subpanels Fig. 4a–d display examples for four different parameter conditions) are depicted in Fig. 4. The spectral excitation patterns calculated for a low carrier frequency of 1000 Hz and a high *f_modA_* of 300 Hz (Fig. 4a) show large differences comparing the excitation pattern elicited by the A SAM tone modulation (Δ*f_mod_* = 0 octaves) to the excitation pattern elicited by a B SAM tone with the same carrier frequency of 1000 Hz but a modulation frequency of 479 Hz (Δ*f_mod_* = 0.7 octaves, 50% threshold of 2-stream responses). In contrast, the spectral excitation patterns elicited by the A SAM tone (Δ*f_mod_* = 0 octaves) with the same low carrier frequency but a low *f_modA_* of 30 Hz (Fig. 4b–d, showing a comparison for different tone patterns) differ only slightly from the excitation patterns elicited by the B SAM tone with a higher modulation frequency corresponding to the perceptually measured 50% threshold of 2-stream responses (threshold Δ*f_mod_* 1.0–1.8 octaves for the different conditions, note that all panels have the same axis scaling). Thus, at an *f_modA_* of 30 Hz the modulation frequency difference at the 50% threshold of 2-stream responses results in much smaller differences between the spectral patterns of excitation for the second SAM tone in the triplets than observed for the condition shown in panel (a) of Fig. 4 displaying simulation results for a condition in which spectral cues are likely to explain stream segregation. A generally smaller amount of excitation is found for abutting tone patterns (125 ms/125 ms, 375 ms/375 ms; Fig. 4c, d) due to the effects of suppression that are included in the model, than for the standard tone pattern with short signals well separated in time (125 ms/375 ms; Fig. 4a, b). For both abutting tone patterns (Fig. 4c, d) the onset peak of the B tone is suppressed by the preceding A tone, the model is in an adapted state, resulting in a similarly small excitation. For the standard tone pattern 125 ms/375 ms, however, characterised by a longer SOA_across_ than the TD resulting in a pause between the A and the B SAM tone, the model has recovered from its adaptation state due to the pause, resulting in a strong onset peak and therefore in a much larger average excitation, depicted in Fig. 4a, b. In conditions with a large Δ*f_mod_* of 4 (in octaves) off-center filters are excited by the sidebands which are in a less adapted state and thus show considerably higher excitation compared to filters close to the carrier frequency.

## Discussion

Human psychophysical studies of auditory stream segregation have focused either on signals providing spectral cues (e.g., [5], [7], [38] or on signals providing temporal cues (e.g., [12], [22]–[24]. The SAM tones used in the present study and in a study on the neural correlate of auditory stream segregation in the avian forebrain [1] are more natural signals providing both types of cues: SAM tones with a low carrier frequency in combination with a high modulation frequency provide mainly spectral cues to the auditory system, whereas SAM tones with a high carrier frequency in combination with a low modulation frequency provide mainly temporal cues (e.g., [3], [13]). The aim of the study was to compare the psychophysical results of the present study with the neurophysiological results obtained from bird forebrain neurons [1] and to discuss the relative importance of spectral and temporal cues in auditory streaming of SAM tones using a spectral excitation pattern model representing the auditory periphery [26]–[27]. In the following we will focus on these topics separately.

### Comparison of psychophysical and neurophysiological results

The present psychophysical study applied SAM tones with parameters comparable to those used for ABA- SAM tone sequences presented to avian forebrain neurons in the neurophysiological study by Itatani and Klump [1]. They presented SAM tones with a carrier frequency ranging from 400 to 6000 Hz, a tone duration of 125 ms and a varying stimulus onset asynchrony (SOA_across_) from 125 to 500 ms. The modulation frequency of the A SAM tone was lower than 160 Hz, whereas the modulation frequency of the B signal was 0.5 to 4 octaves higher than 160 Hz in 0.5 octave steps. In general, Itatani and Klump [1] observed a decrease in spike rate in response to the B signal with increasing modulation frequency. Significantly smaller neuronal activity was observed in response to the B signals at higher modulation frequencies than 1 octave. This result is in agreement with those obtained with comparable SAM tone conditions (*f_c_* = 1000 Hz; *f_modA_*≤100 Hz) in the human psychophysical study presented here indicating a segregated percept for signals at a modulation frequency difference between 0.6 and 1.8 octave (Fig. 1).

In addition, Itatani and Klump [1] recorded fewer spikes when presenting ABA- triplets with a constant TD and a short SOA_across_ of 125 ms than when presenting ABA- triplets with the long SOA_across_ of 250 and 500 ms. They suggested that the reduction in spike rate at the short SOA_across_ indicates a contribution of forward suppression to stream segregation (see also [39]). Already in previous psychophysical (e.g., [5], [7]) and physiological (e.g., [9], [11], [16]–[17]) studies of stream segregation employing pure tones differing in frequency the combined effects of TD and SOA_across_ indicated that forward suppression is likely to contribute to auditory stream segregation. Based on the study by Itatani and Klump [1], we predicted that the presentation of ABA- sequences with the tone pattern having abutting short signals (125 ms/125 ms) should result in a stronger perceptual segregation than the presentation of the standard tone pattern with the same short signals but a longer SOA_across_ of 375 ms. The psychophysical results in the present study matched the predictions based on the assumption that forward suppression is relevant for stream segregation (Fig. 1), since the tone pattern with abutting short signals (125 ms/125 ms) was perceptually more segregated than the standard tone pattern (125 ms/375 ms) with widely spaced short signals. This observation is in agreement with the psychophysical modulation detection experiments of Wojtczak and Viemeister [14] and Wojtczak et al. [15], who observed an effect of forward suppression in the amplitude modulation domain that is comparable to forward suppression of pure tones. In both studies an improvement in the SAM modulation detection with increasing delay between masker and signal has been observed. In addition to the effect of tone pattern discussed above (comparison 125 ms/125 ms to 125 ms/375 ms) two more comparisons of TD and SOA_across_ can be evaluated. Regarding stimuli presented with varying TD the present study either kept the SOA_across_ constant (comparison 125 ms/375 ms to 375 ms/375 ms) or varied SOA_across_ according to the TD presented (comparison 125 ms/125 ms to 375 ms/375 ms). In conditions of SAM tone stimuli of varying TD presented with a constant SOA_across_ we observed an increase in the percentage of a 2-stream percept with increasing TD. These results are in agreement with the results from earlier studies [7], [8], [11] presenting pure tone stimuli. In conditions of SAM tone stimuli of varying TD presented with varying SOA_across_ (TD = SOA_across_) we observed a decrease in the percentage of a 2-stream percept with increasing TD. Similar observations have been made in studies presenting pure tones [5], [7], [10].

Bee et al. [10] investigated the effect of various stimulus parameters on adaptation in starling auditory forebrain neurons in relation to the build-up of auditory stream segregation in ABA- sequences. They found that SOA_across_ had a large effect on adaptation and, therefore, on the build-up mechanism. The observation of build-up in the present psychophysical study is consistent with such a mechanism, showing an increase in the percentage of a 2-stream percept with increased presentation duration from 5 to 15 s.

### Evaluation of the relevance of spectral and temporal cues

In the present study auditory stream segregation can be elicited by ABA- SAM tone sequences, in which A and B signals have the same carrier frequency but different modulation frequencies. Some of the A and B SAM tone conditions that were used should have produced noticeable spectral excitation pattern differences (e.g., A signal: *f_c_* = 1000 Hz, *f_modA_* = 300 Hz; B signal: *f_c_* = 1000 Hz, *f_mod_* = 479 Hz, Fig. 4a). Other of the A and B SAM tone conditions should not have produced noticeable spectral excitation pattern differences forcing the auditory system to rely on temporal cues to discriminate these A and B signals (e.g., A signal: *f_c_* = 1000 Hz, *f_modA_* = 30 Hz; B signal: *f_c_* = 1000 Hz, *f_mod_* = 103 Hz, Fig. 4b). The computational model [26]–[27] applied in the present study includes effects of adaptation and suppression and predicts a lower mean B tone excitation when simulating the response to ABA- SAM tone patterns with abutting signals (TD = SOA_across_; Fig. 4c, d) rather than simulating the response to the standard tone pattern with a silent interval between signals (125 ms/375 ms; Fig. 4a, b).

To our knowledge, the only other study that used SAM signals in a stream segregation paradigm but only provided temporal cues for stream segregation was conducted by Grimault et al. [12] who used broad-band noise as the carrier but otherwise applied similar temporal patterns as in the present study. They presented ABA- sequences with abutting noise bursts of a tone duration of 100 ms resulting in a tone pattern of 100 ms/100 ms. The modulation frequency of the A signal was fixed at 100 Hz and the modulation frequency of the B signal was up to 3 octaves higher. Their results show that stream segregation (more than 50% segregated) can occur solely on the temporal basis due to differences in the modulation frequency of the A and the B signal of 0.9 octaves or more which lies above the discrimination threshold for modulation frequencies (0.14 octaves). Even though the A and B SAM tones presented in the present study provide not only temporal cues, a similar modulation frequency difference (Δ*f_mod_* of 0.6 to 0.8 octaves for carrier frequencies of 1000 and 4000 Hz, respectively) led to stream segregation (applying the same 50% segregation criterion) for the most comparable tone pattern (125 ms/125 ms). Similar to the results of the present study, Grimault at al. [12] found that stream segregation is reduced by a decrease in the modulation depth.

If tones (present study) instead of noise are used as the carrier of SAM the carrier frequency, modulation frequency, modulation depth and the width of the auditory filters will determine whether the auditory system relies mainly on temporal cues (as in the study by Grimault et al. [12]) or exploits predominantly spectral cues or possibly both. The width of the auditory filter is narrower for a carrier frequency of 1000 Hz than for a carrier frequency of 4000 Hz (e.g., [3], [13]). Measuring temporal modulation transfer functions of SAM tones, Moore and Glasberg [13] concluded that at a carrier frequency of 1000 Hz the sidebands of the SAM tone can be clearly detected by the auditory system based on spectral cues at a modulation frequency of 160 Hz or above. Using a range of carrier frequencies, Kohlrausch et al. [3] observed that spectral cues become effective for the detection of modulations at a modulation frequency of about 10% of the carrier frequency. In contrast to the mere detection of modulation in a SAM tone that can be explained by spectral cues, the present study requires comparing SAM tones with different modulation frequency in order to segregate these signals.

To predict the perceptual stream segregation thresholds (50% thresholds of 2-stream responses) for these SAM tones, the present study engaged a computational model [26]–[27] calculating the mean differences between the spectral excitation patterns during the total tone duration elicited by the second SAM tone with a Δ*f_mod_* of 0 octaves (reference) and the B SAM tone with a Δ*f_mod_* of up to 4 octaves in the ABA- sequence. One model scaling parameter was fitted to the results for three SAM tone conditions to the perceptually measured data in which the use of spectral cues was most likely (i.e., at a low carrier frequency and a high modulation frequency of the A signal, see method section) and assuming that a similarly large difference between the spectral patterns of excitation elicited by the A SAM and B SAM signals differing in modulation frequency applies also for the other 50% thresholds of 2-stream responses in which no fit was made.

If the 50% threshold of 2-stream responses predicted by the excitation pattern model is considerably larger (i.e., larger Δ*f_mod_* at threshold) than the perceptually measured threshold (e.g., more than 3 SE above the measured 50% threshold of 2-stream responses; see Fig. 3), it can be concluded that perceptual segregation at the 50% threshold of 2-stream responses is unlikely to rely on spectral cues suggesting the use of temporal cues in those conditions. A clear example is provided by the SAM stimuli conditions with a carrier frequency of 1000 Hz and a modulation frequency of the A signal of 30 Hz for which the spectral excitation pattern model clearly fails to explain the perceptually measured data. Similarly, SAM stimuli with a carrier frequency of 4000 Hz and a modulation frequency of the A signal of 30 or 100 Hz are not likely to be segregated based on differences in spectral patterns of excitation. The closest match was found between the 50% threshold of 2-stream responses predicted by the model and the perceptually measured 50% threshold of 2-stream responses (excluding the three conditions used for fitting the data, in which the match simply results from the fitting) for a modulation frequency of the A signal of 300 Hz, indicating that in such conditions the use of spectral cues is likely. Thus, the computational model calculations suggest that in the present study stream segregation by SAM tones may be predominantly due to temporal cues for the lowest modulation frequency of the A signal of 30 Hz and in most conditions for the modulation frequency of the A signal of 100 Hz, but spectral cues are likely to be involved at the highest tested modulation frequency of the A signal of 300 Hz. These predictions are consistent with previous results on the detection of spectral sidebands of SAM tones discussed in the psychophysical studies mentioned above (e.g., [3], [13]).

In the control experiment, we observed a small increase in the mean percentage of 2-stream responses (3.6%) compared to the mean percentage observed in the main experiment. Furthermore the mean percentage of a 2-stream percept (Fig. 2) in the control experiment shows a larger SEM than the one observed in the main experiment. We reason that this difference of the SEM in the two experiments is due to the different number of subjects tested in the experiments. In the control experiment less than half of the number of subjects than in the main experiment participated, thus increasing the size of the SEM. Since both experiments relied on a different sample of subjects, it could well be that this significant but small difference indicates a possible difference in the subjects' internal criterion or other differences in perception. However, if distortion product would have provided important cues to stream segregation, a decrease rather than an increase in the percentage of a 2-stream percept would be expected. Thus, we conclude that additional spectral cues provided by distortion products do not contribute to the observed thresholds in the main experiment. In agreement with this conclusion, the computational model of excitation pattern differences failed to predict any 50% thresholds of 2-stream responses for the range of modulation frequency differences between the A and the B signal that was tested. The failing of the excitation pattern model suggests that the masking by pink noise prohibited stream segregation of the ABA- sequences based on spectral cues and, therefore, the use of temporal cues is more likely in the control experiment. The pink noise masker itself contributed so substantially to the spectral pattern of excitation that spectral differences being due to modulation frequency differences between A and B signals were considerably reduced so that this pattern difference was smaller than the reference value 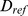 used by the model to estimate the 50% thresholds of 2-stream responses for all conditions presented to the subjects. In agreement with the masking of spectral differences no significant difference was observed for both tested carrier frequencies in contrast to the results of the main experiment.

### Conclusions

Natural signals like sinusoidally amplitude modulated (SAM) tones provide spectral, temporal or both types of cues. The present study evaluated the ability of human subjects to segregate SAM tones having the same carrier frequency but different modulation frequencies. An increasing modulation frequency difference of the A and the B SAM tone in an ABA- sequence induces an increase in the percentage of a 2-stream percept in human subjects (Fig. 1, 2).

This increase is in agreement with the results of the neurophysiological study by Itatani and Klump [1] for comparable SAM stimuli conditions. In agreement with their study we observed the smallest perceptual segregation for the standard ABA- SAM tone pattern with a TD of 125 ms and a SOA_across_ of 375 ms (Fig. 1). The largest percentage of a 2-stream percept was elicited by the tone pattern of abutting short signals (125 ms/125 ms). The observed perceptual effect supports the idea of forward suppression also observed in the neurophysiological study by Itatani and Klump [1].

In general, the perceptual segregation of SAM tones differing in modulation frequency can be triggered either by spectral, temporal or both types of cues. By implementing a computational model based on spectral excitation pattern differences [26]–[27] we could show that for the low carrier frequency of 1000 Hz and high modulation frequencies spectral cues are likely to be used to segregate the SAM tones (Fig. 3, 4), whereas at the high carrier frequency of 4000 Hz and a low modulation frequency of 30 or 100 Hz the excitation pattern model fails to explain the stream segregation thresholds and thus in such conditions stream segregation by temporal cues is a more parsimonious explanation.
